# 3D printed complete removable dental prostheses: a narrative review

**DOI:** 10.1186/s12903-020-01328-8

**Published:** 2020-11-27

**Authors:** Eva Anadioti, Leen Musharbash, Markus B. Blatz, George Papavasiliou, Phophi Kamposiora

**Affiliations:** 1 Department of Preventive and Restorative Sciences, University of Pennsylvania School of Dental Medicine, 240 South 40th Street, Philadelphia, PA 19104-6030 USA; 2 Department of Prosthodontics, National and Kapodistrian University of Athens, Athens, Greece

**Keywords:** Complete removable dentures, Digital dentures, CAD/CAM, 3D printed, Rapid prototyping, Additive manufacturing

## Abstract

**Background:**

The purpose of this paper is to review the available literature on three-dimensionally printed complete dentures in terms of novel biomaterials, fabrication techniques and workflow, clinical performance and patient satisfaction.

**Methods:**

The methodology included applying a search strategy, defining inclusion and exclusion criteria, selecting studies and forming tables to summarize the results. Searches of PubMed, Scopus, and Embase databases were performed independently by two reviewers to gather literature published between 2010 and 2020.

**Results:**

A total of 126 titles were obtained from the electronic database, and the application of exclusion criteria resulted in the identification of 21 articles pertaining to printed technology for complete dentures. Current innovations and developments in digital dentistry have successfully led to the fabrication of removable dental prostheses using CAD/CAM technologies. Milled dentures have been studied more than 3D printed ones in the currently available literature. The limited number of clinical studies, mainly case reports, suggest current indications of 3D printing in denture fabrication process to be custom tray, record bases, trial, interim or immediate dentures but not definitive prostheses fabrication. Limitations include poor esthetics and retention, inability to balance occlusion and low printer resolution.

**Conclusions:**

Initial studies on digital dentures have shown promising short-term clinical performance, positive patient-related results and reasonable cost-effectiveness. 3D printing has potential to modernize and streamline the denture fabrication techniques, materials and workflows. However, more research is required on the existing and developing materials and printers to allow for advancement and increase its application in removable prosthodontics.

## Background

Despite the reduction in the incidence of edentulism in this generation cohort [1], the absolute number of edentulous patients is increasing due to the increase in life-expectancy [2–4]. Complete removable dental prostheses (CRDP) or complete dentures (CD) have been used to rehabilitate patients with complete edentulism for centuries [5]. Those prostheses meet the minimum social and physiological needs of the patients [6] and have not evolved significantly in recent years.

The most commonly used material for fabrication of conventional CRDP has been the polymer polymethyl methacrylate (PMMA) [7]. The material’s relative ease of processing and repair, biocompatibility, and esthetic characteristics have led to increased acceptability by the patients [8]. Nevertheless, PMMA has numerous disadvantages including high polymerization shrinkage, susceptibility to microbial colonization from the oral environment, lack of radio-opacity, allergic reactions mostly due to leaching of the monomer, degradation of the mechanical properties over time and low wear resistance in human saliva. These shortcomings have steered novel materials and manufacturing techniques, both additive and subtractive, to transpire [9, 10].

Contemporary advancements in digital dentistry have started to affect the fabrication of this treatment modality. Digital dentistry has revolutionized the practice of dentistry in many fields since its introduction in the 1980s [11]. In 1994, the first attempt of developing a computer-aided-designed/computer-aided-manufactured (CAD/CAM) system to fabricate a complete removable dental prosthesis emerged [12]. The launch of digital denture construction, however, was marked by Goodacre et al. in 2012 [13]. In this article, a prototype served as an example of the type of program that could be incorporated in the future fabrication of digital dentures. Today, an exponential increase in the number of materials available in the market for fabrication of digital CRDPs is attributed to the ongoing evolution and enhancement of digital technologies [14].

There are two main digital fabrication processes for removable dental prostheses; the subtractive and the additive [15]. With the subtractive method, the denture base is milled from a prepolymerized resin blank. Depending on the system, prefabricated or milled denture teeth are subsequently bonded on the base. Such contemporary systems include Zirkonzahn Denture System (Zirkonzahn, Italy), Ivoclar Digital Denture (Ivoclar Vivadent, Liechtenstein), Vita Vionic (Vita Zahnfabrik, Germany) and AvaDent Digital Dentures Bonded Teeth (AvaDent, USA). Recently, few systems developed a method to mill the denture and the teeth out of a single blank AvaDent Digital Dentures XCL1 and XCL-2, Baltic Denture System (Merz Dental, Germany) and Ivoclar Vivadent Ivotion. The main disadvantage of the subtractive technique is the waste, as a large portion of the blank remains unused and is discarded during this process. Another limitation is the monochromatic and unesthetic teeth, which AvaDent has overcome in their XCL-2 denture by using a unique layering system resulting in polychromatic teeth that simulate the dentin and enamel of natural teeth, providing premium esthetics [16].

Additive manufacturing (AM), also known as 3-dimensional (3D) printing or rapid prototyping (RP), encompasses techniques that fabricate objects layer by layer. 3D printing, despite its relative recent introduction, has shown potential in many fields like engineering and medicine including dental medicine [17]. The available 3D printing systems for complete removable dental prostheses are FotoDenta denture (Dentamid, Germany) and Dentca 3D Printed Denture (Dentca, USA) [15].The limited resolution and reproducibility of the available printers along with their technical constraints have so far posed obstacles in such manufacturing methods of dental restorations [18, 19].

The emerging AM technology is modifying the clinical and laboratory processes of fabricating removable prostheses. The purpose of this paper is to review available literature on 3D printed complete dentures in terms of novel biomaterials, fabrication techniques and workflow, clinical performance, and patient satisfaction.

## Methods

The methodology included applying a search strategy, defining inclusion and exclusion criteria, and retrieving studies; selecting studies; extracting relevant data; and forming tables to summarize the results. Searches of PubMed, Scopus, and Embase databases were performed to gather literature published between 2010 and 2020. The search terms used were “Denture” [Mesh] OR ‘‘Removable Dental Prostheses” OR “Removable Denture” OR “Complete Denture” AND “CADCAM” [Mesh] OR “CAD/CAM” OR “CAD-CAM” OR “Computer Aided Design and Computer Aided Manufacturing” AND “Milled” [Mesh] AND “3D Printed” OR “Printed” AND “Digital Denture” [Mesh].

The inclusion criteria for selection were articles written in English published between 2010 and 2020 on 3D printed dentures, clinical studies and in vitro studies, technique articles that reported workflow, clinical complications or quality assessment with 3D printed dentures. Exclusion criteria included any articles that failed to involve items described in the inclusion criteria or any article that described repetitive data from another included article was excluded. Additionally, articles on 3D printed removable partial dentures (RPD) or partial dental prostheses (PRDP) were also excluded. The search strategy for this review involved 3 stages: reviewing titles, abstracts, and final selection of articles for full text analysis. Articles selected from the database search were sorted independently by 2 reviewers, and any differences in selection were discussed until a consensus was reached. Upon the reviewers’ agreement, articles that did not meet the predetermined inclusion criteria were excluded. Abstracts of the articles selected at the second stage were independently evaluated by the same reviewers, and articles selected for final analysis were obtained in full text. At the third and final stage, the full text of the obtained articles was analyzed.

## Results

### Dental materials for 3D printed denture base and teeth

Currently, there is a very limited number of in-vitro studies evaluating the materials’ properties and accuracy for 3D printing dentures, denture bases and denture teeth. The accuracy of fit between denture base and mucosal tissue is key for the retention of CRDP and long-term success of the prosthesis. Milled CRDPs have been shown to have accurate adaptation compared to conventionally processed dentures [20]. With regards to 3DP dentures, an in-vitro study quantitatively compared their tissue surface adaption against the traditional manual method [21]. A wax pattern of a maxillary complete denture was made on a standard edentulous plaster cast using high-precision 3D wax-printing technology (CAD&3DP), and the fit between the wax pattern and the cast was evaluated quantitatively. There was no statistically significant difference observed between CAD&3DP group and manual manufacturing group for measurements of deviation between the denture tissue surface and the plaster cast model. Therefore, this study suggested that the use of 3D printing manufacturing to fabricate CRDP for try-in appointments when restoring edentulous jaws, appeared to be clinically acceptable.

A recent in vitro study compared the difference in trueness between CAD/CAM milled and 3D printed dentures [22]. The 3DPD group (n = 10) comprised complete dentures fabricated by an RP technique using a 3D printer (RapidShape D30; Rapid Shape GmbH) while the MDG group (n = 10) consisted of milled complete dentures (AvaDent Digital Dental Solutions Europe). The 3DPD group base and teeth were printed together as one unit while the milled group had the based milled and then commercially available teeth bonded to it. The entire intaglio surface and certain regions of interests were selected for analysis. The prostheses were aged by saliva immersion and then wet–dry cycle. The results showed that CAD/CAM milled CRDP was superior in terms of trueness of the entire intaglio surface (*P* < 0.001) at baseline, after immersion in saliva, and after the wet–dry cycle. Intragroup results revealed a significant difference in the trueness of the entire intaglio surface in 3DPD when compared between baseline and after immersion in saliva (*P* < 0.001) and between baseline and after the wet–dry cycle (*P* = 0.003). Therefore, the 3D printed dentures have less dimensional stability over time than the milled ones, but the clinical implication of those changes is not evaluated yet.

Contrary to that, another study evaluated the accuracy and surface resolution of denture bases fabricated by three methods: injection molding, milling, and rapid prototyping using surface matching software. The results comparing the fit accuracy between the cast and the maxillary complete denture base were evaluated on the second upper premolar and the second upper molar regions crossing the midpalatal suture, showing relatively high deformation in the conventional method due to polymerization shrinkage and internal stress. The mean value of discrepancies, however, was the lowest in the RP method, followed by that in the milling method and the injection molding method [23].

The clinical performance of a prosthesis is limited by the mechanical properties of its materials. During mastication, dentures are subjected to flexural stress creating internal stresses. These in return cause cyclic deformation of the polymer base, resulting in crack formation and eventually fracture. In a study on the evaluation of flexure strength and surface properties of prepolymerized CAD/CAM PMMA based polymers, used for digital 3D printed complete dentures, the flexural strength (FS), and hydrophobicity of PMMA-based CAD/CAM polymers was higher in the CAD/CAM PMMA-based polymers compared to the conventional heat-polymerized PMMA, whereas the CAD/CAM PMMA-based polymers had similar surface roughness values to the conventional PMMA [24]. Similar results with regards to high flexural strength of milled resins are found in recent studies [25, 26]. Specifically, Prpić et al. [26] compared flexural strength and surface hardness between three CAD/CAM milled (IvoBase CAD, Interdent CC disc PMMA, and Polident CAD/CAM disc), one 3D-printed (NextDent Base), and one polyamide material (Vertex ThermoSens) for denture base fabrication. The 3DP resin had statistically significant lower flexural strength than the other materials tested with a range of 60–85 MPa. The authors suggested that it was within clinically acceptable limits (65 MPa) based on ISO standards. Surface hardness was also amongst the lowest. The authors concluded that milled or conventionally processed dentures are currently superior to 3D printed ones.

A shortcoming of PMMA is the great susceptibility to microbial colonization from the highly contaminated oral environment. Several studies recognized the incorporation of approximately 5% weight of TiO_2_ into acrylic denture base structure has an antibacterial effect [27–29]. This amount however can cause internal decomposition and weaken the material. 3D printing manufacturing of digital dentures allows the development of new biomaterials with improved properties. This issue was addressed by Totu et al. [30], by using of a nanocomposite PMMA 0.4% TiO_2_ nanoparticles, which inhibit the growth of Candida Scotti strain in standard conditions, according to the toxicity control method (DHA).

With 3D printing, the build direction (layer orientation) affects the mechanical properties of the dental restorative material [31]. This is due to the nature of incremental layers in additive manufacturing technology, which may initiate crack propagation and result in a structural failure of the printed material. In an in vitro study, layer orientation was found to affect the compressive strength of 3D-printed composite material. The material printed vertically with the load perpendicular to the layer orientation exhibits a higher compressive strength than a material printed horizontally [32]. Also, it is important to understand that the bond between the layers is weaker than that within the layer. This is explained by the amount of residual stresses and porosities that accumulate during UV polymerization and material shrinkage [33].

Choi et al. [34] compared fracture toughness and flexural bond strength between three types of denture-base resins (DBRs), heat cure, CAD‐milled, and 3D printed, and four different types of commercial denture teeth (Unfilled PMMA, double cross‐linked PMMA, PMMA with nanofillers and 3D printed resin teeth). All specimens were surface treated, bonded, and processed according to manufacturer's instructions. A 4‐point bend test, using the chevron‐notched beam method, was performed. The results revealed that teeth bonded to heat‐cured denture-base resins produced the highest fracture toughness. Teeth bonded to CAD/CAM and 3D printed DBRs showed significantly lower bond strength. The study suggested that despite the increasing popularity of CAD-milled and 3D printed materials, heat-cured denture base resins still produce the highest bond strength to various types of denture teeth. 3D printing of denture teeth is a novel method and uses newly developed materials. DENTCA, for example, developed a resin material specifically for 3D printing denture teeth. Chung et al. [35] compared chipping and indirect tensile fracture resistance of 3D printed resin denture teeth to prefabricated resin denture teeth. 3D printed resin teeth had comparable fracture resistance to some of the conventional prefabricated denture teeth. Cha et al. [36] evaluated the wear resistance of 3D printed resin denture tooth opposing zirconia and metal. The wear behavior of the printed denture tooth resin was comparable to that of prefabricated denture teeth. With regards to improving wear resistance, other authors have incorporated designing of metal functional cusps separate from the denture [37]. The separated functional cusp file was printed in metal, and the metal cusp was bonded to the PMMA resin-printed denture base.

### Manufacturing techniques and workflows for 3D printed dentures

Efficiency is one of the driving factors towards workflow optimization through digital dentistry. Similar to fixed dental prostheses, removable prostheses fabrication was digitized first in the dental laboratory with scanning of the impressions or casts, digital tooth set up, and milling or printing of the trays/record bases or even final prosthesis. This process allowed for faster turnaround times and design storage in case of prostheses loss or fracture but did not necessarily affect the clinical appointment sequence or workflow. The first implementation of digital denture workflow, in a clinical setting, was the single appointment for preliminary/final impressions along with jaw relation records and teeth selection. One appointment that could be eliminated is the try-in appointment due to the potential virtual evaluation of that step with a software. Lastly, the number of needed adjustments may be reduced as the accuracy of digital dentures is claimed to be superior to the conventional ones [20]. Consequently, the number of clinical appointments could be reduced from more than five, depending on number of necessary adjustments, to three.

Initial reports describing the CAD/CAM fabrication of CRDPs less than a decade ago, demonstrated several advantages. The two systems that were commercially available first for fabrication of digital complete dentures were Avadent and Dentca [38, 39]. Avadent uses laser scanning and proprietary software to arrange the denture teeth and design the bases. Dentca, on the other hand, uses computer software to produce virtual maxillary and mandibular edentulous ridges, arrange the teeth, and form bases. The dentures in AvaDent are milled from prepolymeried pucks of resin while those of Dentca were initially fabricated with a conventional processing technique.

After digital CRDPs grew in the dental market, more CAD/CAM systems surfaced each year [14]. The different denture systems can be compared based on number of dental visits needed, assessment and registration of vertical dimension, determination of dental or facial midline, registration of maxillomandibular jaw relation, and try-in options [40]. According to manufacturers’ recommendations, the number of patient visits, including try-in appointments, is four with Wieland digital denture, three with both AvaDent digital dentures and Whole You Nexteeth, and two with Baltic Denture System. With regards to the assessment of the occlusal vertical dimension, all systems rely on the dentist to take measurements of the esthetic height of the lower face. Wieland digital denture system provides individual trays, milled in the correct occlusal vertical dimension, for bite registration. With AvaDent digital denture, an anatomical measuring device is used while for WholeYou next teeth, a bite registration pin is extruded. Individually relined Baltic denture keys are used with Baltic denture system. To determine the occlusal plane, Wieland digital denture uses a UTS CAD transferring arch. AvaDent digital dentures require a properly adjusted anatomical measuring device or wax rim. With Whole You Nexteeth, the determination of the occlusal plane is done digitally. For the determination of dental midline, all systems rely on the dentist to mark the midline except for Whole You Nexteeth, where the midline is determined digitally. Regarding the registration of maxillomandibular jaw relation, Wieland, AvaDent digital dentures and Whole You next teeth use gothic arch registrations while Baltic denture system uses relined Baltic denture keys into centric relation. Finally, different systems provide different try-in options. Wieland digital dentures provide milled white PMMA monolithic CRDP, AvaDent digital denture wax or milled PMMA monolithic CRDP, WholeYou Nexteeth a 3D printed white acrylic polymer, and Baltic Denture system allows adjustments to the Baltic denture keys.

All the above systems are based on milling technology. The first 3D printed denture was developed by Dentca in 2015. In their workflow, the dentist can make either a digital or conventional impression along with jaw relation records and send them to the dental laboratory. A CAD design software is used to design the denture base with the teeth in occlusion. There is the option for a printed try-in denture, where adjustments can be made clinically by grinding the acrylic and then rescan. The final denture and teeth are printed separately and then bonded together. Moreover, as the cost-effectiveness of tabletop dental printers and open source software is improving, dental clinicians and laboratories have the ability to print CRDPs in-house. However, due to the recent introduction of 3D printed dentures, the available literature is limited to innovative “proof of concept” reports on the implementation of 3D printing in removable prosthodontics. There is currently no evidence on indicated usage, software, sequence or workflow available.

As dentistry is moving towards a fully digital workflow, intraoral scanning is being considered for replicating soft tissues. A case report that used intraoral scanning for initial data acquisition showed the shortest pathway from data acquisition to the final denture delivery within a fully digital workflow in only two appointments [41]. However, this approach did not include try-in session for evaluation of the final esthetic outcome and, most importantly, since there is no border molding done, the retention for the final prosthesis was poor. The authors modified their technique by the implementation of digital relining (DR) where a trial denture was milled. This was used to reline the intaglio surfaces intraorally and to perform an esthetic evaluation. The relined trial denture was then digitized, teeth set-up was adjusted based on the evaluation, and the final prostheses were printed. Similarly, other authors have mainly recommended scanning the existing maxillary and mandibular CRDPs, 3D printing them, and using them as a custom tray [42–44] or trial dentures [45] for conventional workflow.

In terms of these limitations, the in-office additively manufactured interim CRDP following a digital workflow was proposed [46]. The workflow began with an intraoral scan and a maxillomandibular occlusal record, which were exported in a standard tessellation language (STL) file. Next, a CAD software was used to define the existing mandibular plane, followed by a diagnostic tooth arrangement in the same CAD software. The definition of denture base extension on the virtual edentulous ridge was done and a virtual denture base of 3 mm thickness was created. The approved design of the virtual diagnostic tooth arrangement and denture base were exported as 2 individual STL files and imported into a support-and-build preparation software. An in-office 3D printer was used to fabricate the denture base with soft-tissue-colored and the diagnostic tooth arrangement with tooth-colored photopolymerizing resins. After polymerization in a light-polymerizing unit, the diagnostic tooth arrangement was luted to the denture base with a soft-tissue-colored photopolymerizing resin. Finally, the interim CRDP was relined with soft reliner (Coe-Soft; GC America Inc) to facilitate insertion and improve retention.

Another indication for 3D printing has been the immediate CRDP. Neumeier et al. [47] proposed that through the digital process, a single digital design and a definitive digital record could be created, which can be used to fabricate the immediate digital denture and surgical reduction guide for alveoloplasty. Digital immediate dentures can be relined with the same process as conventional dentures. The definitive digital dentures can be fabricated with a reline impression and new centric relation record, using the existing digital immediate denture without additional clinical procedures. Providing patients with 3D-printed immediate or interim dentures seems to be a viable treatment modality considering the current limitations.

With respect to clinical workflow and steps, the assessment of occlusal vertical dimension, maxillomandibular relationships, lip support, and maxillary incisal edge position may become challenging with the digital CRDP workflow, especially for novices [48, 49]. In addition, patient input is minimal with elimination of try-in, and current material and laboratory costs are higher than those of the traditional methods. Moreover, digital dentures require system-dependent equipment, such as trays, materials, software and specific training. For clinicians, it is important to understand which technique matches their practice, expertise, and training to implement new workflows. As companies and techniques expand, it may be wise to consider open digital systems [50]. This allows dental professionals to tailor growing digital technology to individual needs without affecting clinical excellence or practice efficiency.

From a laboratory standpoint, several errors in reviewing the digital design of tooth set-up virtually have been reported and careful assessment is recommended with usage of a checklist, at least at the beginning [51]. Lastly, an important limitation of this digital denture workflow is the difficulty to achieving a balanced occlusion. Currently, only a lingualized centric occlusion can be achieved, while balanced occlusion in protrusive and lateral movements is still under research [52].

At this time, a combination of conventional impressions and maxillomandibular relationship procedures with recent CAD/CAM production and processing techniques may allow clinicians to apply the advantages of both methods to achieve optimum results. However, this may require an additional appointment for the fabrication of custom impression trays and record bases after the initial impressions. At this initial stage of 3D printing technology, the balance between conventional and digital workflows may be required to maintain high clinical standards with incorporation of contemporary techniques.

### Clinical performance and early patient-related outcomes for 3D printed dentures

A significant goal of incorporating new technologies into the dental practice is to provide better treatment solutions for the patients. There are only a few clinical studies with small sample sizes either case reports or pilot prospective cohorts, mainly on milled digital dentures, The retention with milled complete denture bases from prepolymerized poly(methyl methacrylate) resin is significantly higher than that with conventional heat-polymerized denture bases [53]. Esthetics appears to be the limiting factor when evaluating the clinical outcomes of a two-appointment process for digital dentures [54, 55]. Generally, more adjustment appointments were necessary than indicated by the manufacturers [55, 56] while relining has been reported to be required in as much as 40% of the digital dentures [57].

The lower number of appointments required for their denture fabrication as well as the good initial results have led to the finding that patients are, generally, satisfied with a digital denture treatment [56, 58, 59].

There is one prospective clinical study including thirty-five fully edentulous patients that received 3D printed CRPDs with a three-appointment partially digital workflow [60, 61]. First and second appointments included conventional preliminary and final functional impressions, along with maxillo-mandibular relations records and tooth selection. After the casts were poured, they were digitized, and a software was used to design the tooth set-up. A complex nanocomposite (0.4% TiO_2_ nanoparticles reinforced PMMA) was used with Digital Light Projection Manufacturing (DLPM), using EnvisonTEC Perfactory® 3D printer to manufacture the complete dental prosthesis [30]. The authors evaluated retention and stability of the 3DP CRDP at 1 week, 5 months, 12 months and 18 months post denture insertion by two experienced prosthodontists using the modified Kapur index (MKI). A significant improvement in denture retention and stability was noticed for both maxillary mandibular dentures compared to the dentures the patients had previously (*P* < 0.05). The maxillary prostheses were ranked higher than the mandibular ones and that result was maintained during the follow-up period. This result is consistent with the need of implant retained overdentures for severely resorbed mandibles. Additionally, the participants were asked to complete a Visual Analogue Scale (VAS) to evaluate their satisfaction as well as an Oral Health Impact Profile for Edentulous Patients (OHIP-EDENT) to assess their self-perception on their oral-health-related quality of life. The questionnaires were completed prior to the treatment (baseline, T0), at 1-week post denture insertion (T1), and at 12 (T12) and 18 months (T18) follow-up. Significant reductions in OHIP-EDENT scores for the maxilla, mandible, both restored arches, and for the overall treatment group were registered at the 1 week and 12- and 18-month follow-ups (*P* < 0.05). Statistically significant improvement in satisfaction was found in all questions asked. However, the lowest mean value for the 3D-printed dentures was registered at 18 months for aesthetic evaluation. This was explained due to the color changes of acrylic resins in the oral cavity, as a result of slow water absorption.

From the clinician’s standpoint, the learning curve of the new technologies along with its current limitations in accuracy and streamlined workflow have shown conflicting results [55, 56, 58, 62]. Experienced clinicians have shown more skepticism with the implementation of digital denture processes than predoctoral students. This may be explained due to the lack of exposure to digital technology during their early career as compared to the students/younger clinicians today for whom technology is integral part of life. On the other hand, the experienced clinicians have much higher expectations in clinical performance and are not willing to compromise the quality of treatment or fundamental prosthetic principles, whereas novices may not be able to see the limitations of a technique due to lack of experience and long-term evaluation of complications.

### Parameters that will drive the swift to 3D printed dentures

With a projected increase of edentulous patients in the future, the need for CRDPs as a treatment modality is recognized. For computer-engineered removable prostheses, a worthwhile consideration is their economic implications as compared to traditional approaches. The decrease in chairside, laboratory and overall working time for dentists, technicians and patients is the main factor when assessing cost versus time. The time saved by less human involvement, virtual teeth arrangement, and ability to effortlessly store and reproduce prostheses should be viewed against the increased cost of milling machines and accrual of additional required equipment such as intraoral scanner or proprietary custom trays. A study that was designed in an academic setting stated that despite the initially much higher costs of the materials used to fabricate the digital denture protocol, overall it was determined to be a less costly method of producing CRDP in terms of clinical chairside time and laboratory costs [63]. Meanwhile, tabletop 3D printers are much less expensive than a milling center and could be afforded by individual dentists and dental laboratories to offset some of the costs that are currently preventing broad digital denture implementation.

When discussing reducing time to improve efficiency, it is important to note that quality should not be compromised. A clinical step considered for elimination with the digital workflow is the try-in appointment. Although digital systems allow for virtual evaluation of the esthetic analysis, this has not yet been proven to be a reliable and adequate replacement for the clinical evaluation of esthetics and phonetics with the patient’s input. Compromising on the esthetic evaluation will lead to patient dissatisfaction and/or remakes. Therefore, most published reports recommend clinical try-in for a reliable evaluation. This adds more to the cost and time but may, ultimately, provide better results.

When in-house 3D printed denture fabrication becomes streamlined with a two-appointment process that includes reliable virtual esthetic and functional assessment, the amount of waste from conventional or milled workflows will be decreased. The ability to treat edentulism locally but also in lower-income areas or nations, where skilled dental technicians are scarce, will increase and the contribution to public health will be significant considering the comorbidities associated with edentulism [4, 64].

Finally, when reflecting on the transformation of dental education with the incorporation of blending learning as well as the generational inclination of today’s dental students with technology, there are initial studies that place the education of digital dentures high in the dental student preferences in a predoctoral setting [58]. The study showed that the digital process was equally effective and more time-efficient option than the conventional process of prosthesis fabrication in the predoctoral program. Another cross-section study that compared predoctoral to postdoctoral students fabricating digital dentures showed that the mean number of appointments needed to insert the prostheses at the predoctoral level was 2.33 (95% CI 2.13–2.53) and 2.45 (95% CI 2.15–2.76) at the postdoctoral resident level, both higher than the 2-appointment solution claimed by the company [55]. The reasons for the additional appointments to insert the prostheses were consistent with other reports and included esthetic or phonetic patient dissatisfaction, lack of retention, incorrect occlusal vertical dimension or centric relation as well as operator required teeth try-in evaluation.

An online survey sent to all of the 50 program directors of postdoctoral prosthodontics programs across the United States revealed that all program directors were aware of current trends in complete denture fabrication using CAD/CAM technology but only 10% or less of complete denture cases are currently processed using the CAD/CAM technology, at either the post- or predoctoral levels [65]. However, plans to add digital denture fabrication into their curricula within the next 1 to 4 years were stated in their responses.

## Conclusions

Current innovations and developments in digital dentistry have successfully led to the fabrication of removable dental prostheses using CAD/CAM technologies. 3D printing has the potential to modernize and streamline the denture fabrication techniques, materials and workflows. Current limitations include elimination of try-in appointment without reliable virtual esthetic evaluation, lack of retention with printed polymers requiring reline for clinical acceptability, inability to balanced occlusion that may compromise denture stability or potentially affect bone resorption and long-term color instability that leads to esthetic deterioration. Presently recommended usages for 3D printed complete dentures are interim or immediate dentures as well as custom tray or record base fabrication for conventional workflows. Well-designed clinical studies are needed to scientifically prove the claimed advantages of this technology.

## Data Availability

The material collected for this narrative review is available upon reasonable request.

## Abbreviations

AM: Additive manufacturing
CAD/CAM: Computer aided design/computer aided manufacturing
CRDP: Complete removable dental prosthesis
PMMA: Polymer polymethyl methacrylate
RM: Rapid manufacturing
RP: Rapid prototyping
3D: Three dimensional
